# CCPD under sparsity and low-rank constraints: multi-frequency dynamic functional network connectivity analysis in schizophrenia

**DOI:** 10.3389/fnins.2026.1775687

**Published:** 2026-05-08

**Authors:** Li-Dan Kuang, Yi-Wen Liu, Ting Tang, Wenjun Li, Jin Zhang, Weijun Liang

**Affiliations:** 1 School of Computer Science and Technology, Changsha University of Science and Technology, Changsha, Hunan, China; 2 Affiliated Changsha Central Hospital, Hengyang Medical School, University of South China, Hengyang, Hunan, China

**Keywords:** Coupled canonical Polyadic decomposition (CCPD), dynamic functional network connectivity (dFNC), frequency band, low-rank constraint, schizophrenia

## Abstract

This study aims to jointly extract group-shared connectivity patterns and group-specific temporal and frequency information from multi-frequency dynamic functional network connectivity (dFNC) tensors of healthy controls (HCs) and schizophrenia patients (SZs) using a coupled canonical polyadic decomposition (CCPD) approach. Based on 145 subjects (71 SZs and 74 HCs) from the COBRE dataset, multi-frequency dFNC tensors were constructed via group independent component analysis and a filter-banked connectivity framework. A novel sparse and low-rank constrained CCPD (SLRCCPD) model was proposed to decompose the dFNC tensors, incorporating L1-norm regularization to enhance significant connections and nuclear norm-based low-rank approximation to improve clustering quality. The results revealed significant connectivity differences between SZs and HCs within auditory, somatomotor, cognitive control, visual, and cerebellar networks across five shared dynamic modules. Clustering of group-specific time-frequency weights showed that SZs had significantly higher fractional and dwell time in State 3 at both low- and high-frequency bands, along with fewer state transitions across all bands compared to HCs. The proposed SLRCCPD framework effectively captures abnormal multi-band dynamic functional connectivity in schizophrenia, providing a new computational tool and empirical pathway for investigating brain network dynamics and mechanistic studies of the disorder.

## 1 Introduction

Functional network connectivity (FNC) based on multi-subject fMRI data describes the statistical or correlation between distinct brain networks, typically extracted by group independent component analysis (group ICA), to represent their functional interactions with cognitive abilities (Motlaghian et al., 2023). Moreover, the brain networks are dynamic even when resting, with notably temporal shifts and intensity of functional connections probably happening within a few minutes or less. This implies that the dynamic functional network connectivity (dFNC) of the brain networks varies with time (Preti et al., 2017). The sliding window analysis is one of the widely-used dFNC analysis methods which first divides the subject-specific time courses extracted by group ICA into a series of overlapping rectangular or conical windows and then calculates the dFNC values between two different independent networks for each window (Allen et al., 2014). The dFNC measurements include the Pearson correlation coefficient, inverse covariance matrix, Spearman correlation analysis, shared trajectory weighted average, and among others (Nomi et al., 2016; Faghiri et al., 2021; Liao et al., 2024a; Faghiri et al., 2020). After calculating the dFNC matrices, the clustering state analysis or the fuzzy meta-state analysis is generally performed to assess reoccurring dFNC states or compute high-level state-space metrics (Fu et al., 2018). An increasing number of temporal dFNC analyses based on time courses have been verified to identify significant and interesting dynamic patterns in brain connectivity and provide insight into how the brain functions and cognitive processes (Fu et al., 2018). Therefore, temporal dFNC analyses have been widely applied to common brain diseases (such as Parkinson's disease Hancu et al., 2019, schizophrenia Sakoǧlu et al., 2010, major depression Long et al., 2020, Alzheimer's disease Jack et al., 2008, autism spectrum disorder Yang et al., 2022) and the biological gender and intelligence prediction (Sen and Parhi, 2020). For example, the dFNC analysis in Sakoǧlu et al. (2010) verified that less segregated motor, sensory, and cognitive processes, as well as less segregated default mode network activity for patients with schizophrenia (SZs) than healthy controls (HCs) when executing tasks.

In reality, the window-sliding method of dFNC generally has two shortcomings: the window size effect and the low-pass nature which cannot capture the full frequency range of dFNC (Faghiri et al., 2021). The window-sliding method also acts as a low-frequency filter (Faghiri et al., 2021). Moreover, the majority of dFNC studies focus on low-frequency bands (such as 0.01–0.08Hz) which are related to neural oscillations (Shi et al., 2023). However, the human brain network which is made up of billions of linked neurons is a complex dynamic system that spontaneously generates a large number of oscillation waves (Shi et al., 2023). As a result, more and more studies have begun to investigate the functional connectivity of fMRI data in several different frequency bands. Specifically, the dynamics of functional network integration and segregation in different frequency bands were revealed (Thompson and Fransson, 2015). Wang et al. (2018) found a frequency-dependent regulation of the right anterior insula. To examine the network architecture changes in different frequency bands, Kajimura et al. (2023) separated the blood oxygen level-dependent (BOLD) signal into four frequency bands (ranging from 0.007–0.438 Hz) and used a clustering technique for each band. Luo et al. (2020) showed that dynamic functional connectivity (dFC) strength among different regions of interest (ROIs) in schizophrenia patients (SZs) varies by frequency bands. Faghiri et al. (2021) proposed a unified filter bank connectivity approach to calculate the static and dynamic FNC analyses throughout the whole frequency range. This method discovered some weak states in low frequencies that cannot be recorded using typical window-based approaches (Faghiri et al., 2021). Moreover, compared to HCs, SZs spent more time in higher frequency states (Faghiri et al., 2021). The multimodal connection between structural MRI and FNC states derived via this unified filter bank connectivity approach showed that functional connectivity in SZ-dominant states of low-frequency and high-frequency is significantly linked to gray matter volume in various regions of the frontal and temporal cortices (Duda et al., 2022). In addition to the frequency-dependent functional connectivity analyses, the time-frequency analysis of fMRI data is also widely studied and can capture the dynamic changes of brain activity in both the time and frequency domains, providing more comprehensive brain functional connectivity information than traditional static analysis and improving the understanding of brain dynamics (Chang and Glover, 2010; Yaesoubi et al., 2016; Belhaouari et al., 2023). A sparse non-negative tensor decomposition method of muti-subject fMRI data estimated frequency-specific co-activation patterns which obtained the main differences between patients with Parkinson's disease and HCs in the 0.04–0.1 Hz frequency band of the basal ganglia (Hu et al., 2021).

The dFC between ROIs or dFNC between independent component networks (ICNs) matrices of all sliding windows inherently can be formed as a three-way tensor (ROIs × ROIs × windows for dFC and ICNs × ICNs × windows for dFNC). Considering the multiple subjects, the multi-subject dFC and dFNC matrices can be described as four-way tensors (ROIs × ROIs × windows × subjects for dFC and ICNs × ICNs × windows × subjects for dFNC). Tensor decomposition methods can well preserve the structure information of the higher-way tensor and have shown excellent potential for extracting significant connectivity, time-varying weights, and subject intensities to distinguish the HCs and brain disease patients. The prominent tensor decomposition methods include canonical Polyadic decomposition (CPD) and Tucker decomposition. Gabrielson et al. (2024) proposed a mode coresets-based Tucker decomposition that selects weighted subsets from tensor modes to approximate the full tensor, enabling both computational efficiency and interpretable feature selection in fMRI functional connectivity analysis. Glomb et al. (2021) used CPD to decompose the three-dimensional dFC tensor (ROIs × ROIs × windows) of the single subject into a set of brain regions that share similar temporal dynamics and corresponding time courses. This revealed the temporal dynamics between brain region sets and formed different functional connectivity patterns. The CPD and Tucker decomposition decompose the four-way or three-way dFC tensors into connectivity, temporal, and subject factors (Mokhtari et al., 2019). Moreover, the results of the four-way dFC tensor have higher region connectivity than those of three-way dFC tensor. Sen and Parhi (2020) proposed an orthogonal CPD model to decompose the dynamic connectivity matrix sequence into mutually shared orthogonal connectivity components, shared time-varying weights, and subject-specific intensities, and then investigated the estimated subject factors to predict biological sex and intelligence. Xiao et al. (2022) proposed a spatiotemporal CPD decomposition framework for the three-way dFC tensor (ROIs × ROIs × windows of all subjects). The L1-norm regularization on spatial dynamic modules and manifold regularization on time-varying weights prevent model overfitting and smooth time-varying weights, respectively (Xiao et al., 2022). As such, the whole-brain dFC tensor is decomposed into spatial dynamic modules and corresponding time-varying weights, to reveal the spatial and temporal structure of brain dynamics (Xiao et al., 2022). Kuang et al. (2024) proposed an improved sparse low-rank CPD (SLRCPD) spatiotemporal framework applied to the three-way dFNC tensor (ICNs × ICNs × windows of all subjects). SLRCPD decomposes the dFNC tensor into dynamic spatial modules and corresponding time-varying weights. SLRCPD adds L1 regularization on the dynamic spatial module to extract significant connections and the nuclear norm to provide low-rank and sparse time-varying weights, which improves the following brain state clustering analysis (Kuang et al., 2024). The results of temporal states revealed significant differences between HCs and SZs and high classification accuracy based on time-varying weights was obtained. These findings demonstrate that tensor decomposition can simultaneously extract the spatial and temporal dynamic features for well-interpreting dFNC and dFC analyses.

Coupled canonical Polyadic decomposition (CCPD) is a joint CPD that has a higher representation capacity than CPD. It can decompose several tensors in one or more dimensions that differ in other dimensions. Therefore, CCPD has shown advantages in biomedical data fusion (Borsoi et al., 2023; Kuang et al., 2023), array signal processing (Zheng et al., 2021), hyperspectral super-resolution (Xu et al., 2020), multistatic MIMO radar target localization (Liao et al., 2024b), and so on. It necessitates that one or more factor matrices be shared between multiple datasets, although other factor matrices from distinct datasets may differ under specific situations (Davidson et al., 2013), and the coupled matrix decomposition with shared and different components is unique (Srensen and Sidiropoulos, 2020). Since temporal variability is greater than spatial variability, Kuang et al. (2023) proposed a CCPD under spatial reference and orthonormality constraints to extract shared spatial maps, group-specific time courses, and subject intensities from complex-valued multi-group fMRI data. Liu et al. (2022) utilized a dual coupled non-negative tensor decomposition model to separate two adjacency tensors with dimensions of time, frequency, connectivity, and subject (Tang et al., 2021; He et al., 2018). To minimize computational complexity, a low-rank approximation was used, and the model was optimized using a fast hierarchical alternating least squares method (Wang et al., 2026). The resulting spectral factor and adjacency factor were grouped to investigate oscillatory disconnected networks. For multi-frequency analysis, the temporal window and frequency band array are merged into one dimension, and the resulting reshaped 3D tensor may be decomposed by CCPD into common spatial components, multiple sets of time-frequency weights, and numerous sets of subject intensities. Clustering methods, principal component analysis (PCA), and ICA can then be used to extract additional dynamic brain states (Preti et al., 2017).

In this work, we develop a new CCPD framework under sparsity and low-rank approximation and construct group-specific three-way dFNC tensors to examine the dynamic temporal and spatial differences between HCs and SZs at various frequencies. The primary contributions of this paper are listed as follows:

1) Group ICA on 145 participants (71 SZs and 74 HCs) in the Center for Biomedical Research Excellence (COBRE) dataset with a high model order of 100 was first conducted, then seven ICNs from 41 components of interest (ICs) were identified, and finally the sliding-windowed Pearson correlation values of time courses and filtered the values with 3 different frequency bands are calculated. As such, two three-way dFNC tensors (ICN pairs × (windows × bands) × subjects) by concatenating the temporal windows and frequency bands for HC and SZ groups are constructed.
2) Considering the higher inter-subject temporal and frequency variability than spatial variability, we assume that all subjects share connectivity of spatial ICNs but have different weights of temporal windows and frequency bands and subject intensities for different groups. CCPD method under sparsity and low-rank constraints is proposed to jointly separate the three-way dFNC tensors of HC and SZ groups into the shared connectivity loading matrix, the group-specific time and frequency weights, and the group-specific subject intensities. The sparsity constraint using L1 regularization on connectivity dimension can extract more significant connectivity. To enhance the ensuing cluster analysis, the nuclear norm is used for a low-rank approximation of group time-frequency weights.
3) The between-ICN and within-ICN connections of the five shared dynamic connectivity modules were thoroughly measured. The sensorimotor (SM) and default mode network (DMN) contribute significantly to the majority of connections in dynamic modules 1, 2, and 5, which have positive values. In dynamic modules 3 and 4, on the other hand, the leading component connectivity is negative. Additionally, the between-ICN connections of group-specific dynamic connectivity modules are also evaluated and the majority of HC and SZ linkages display opposing connectivity values in the group-specific dynamic modules 3 and 4.
4) The various time states of the three frequency bands are also derived by using the well-known k-means clustering algorithm on the subject-specific time-varying weights of the five dynamic models. To investigate statistically significant group-specific temporal differences, we used the Mann-Whitney U-tests on two distinct states. The findings demonstrate that the duration of the time (DT), the fractional time (FT), and the number of state transitions (NT) of the three SZ and HC states in various frequency ranges differ from one another. The FT and DT values of SZs in state 3 at low-frequency band 1 and high-frequency band 3 are significantly higher than those of HCs, while SZs present a significantly lower NT values than HCs at all three frequency bands.

The rest paper is organized as follows: Section 2 describes the resting-state fMRI data of COBRE dataset used in this paper and the methods of constructing the multi-subject multi-frequency dFNC tensors of HC and SZ groups, the proposed CCPD under sparsity and low-rank constraints, the shared and group-specific dynamic connectivity modules analyses and the temporal brain state analysis at different filter bands; Section 3 gives the results; Section 4 concludes the conclusion and discussion.This work extends our previous conference presentation by incorporating sparsity and low-rank constraints into the CCPD framework and conducting a more extensive frequency-band analysis (Kuang et al., 2025).

## 2 Material description of the envelope predominantly model

**Figure 1:**
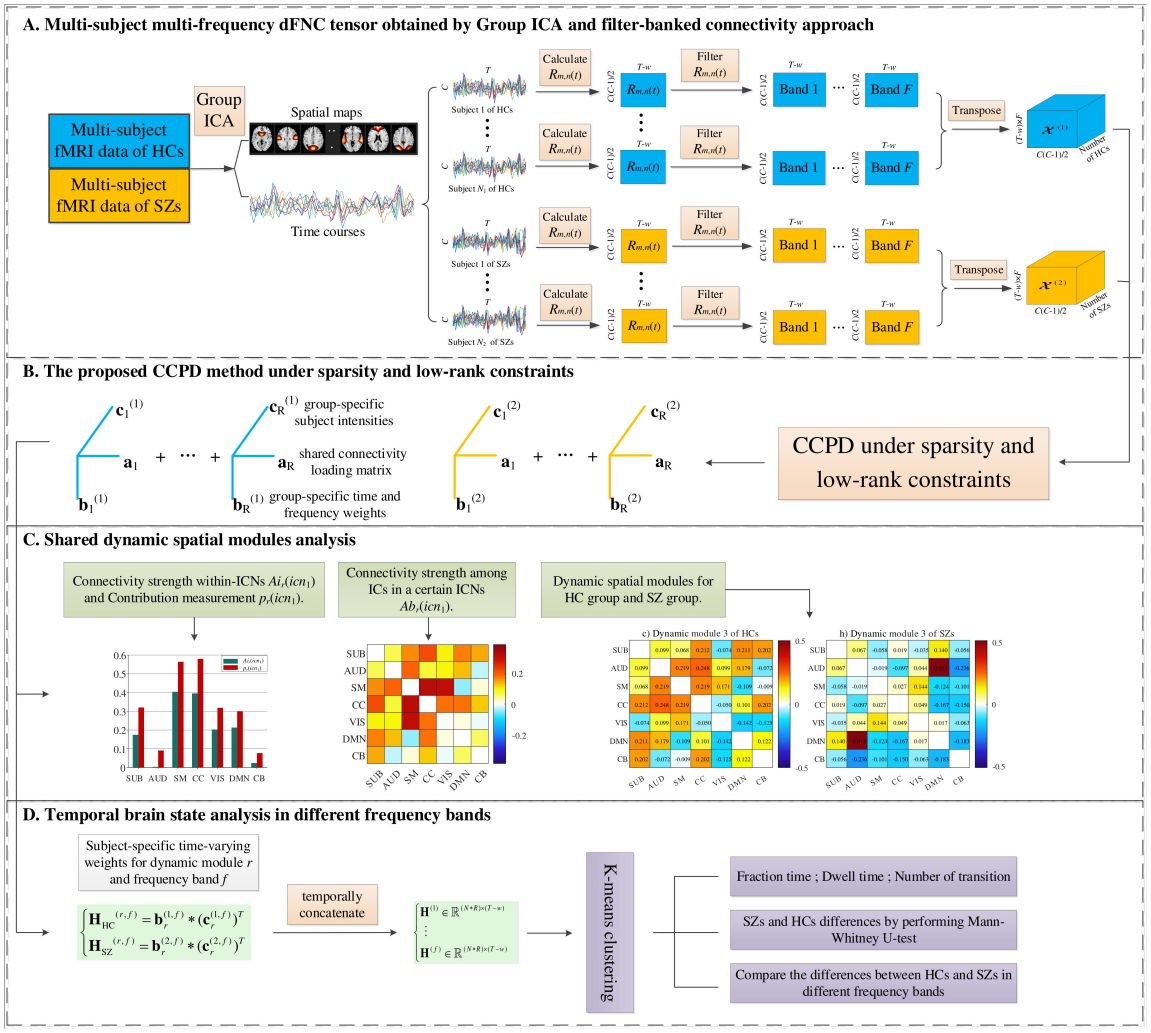
Workflow of the proposed method. The figure shows four stages. **(A)** First, the ICs of interest are extracted by group ICA, and then the multi-subject multi-frequency dFNC tensors of HCs and SZs are constructed based on the filter-banked connectivity approach. **(B)** The proposed CCPD method under sparsity and low-rank constraints is used to decompose the dFNC tensor into a shared connectivity loading matrix, group-specific time and frequency weights, and group-specific subject intensities. **(C)** Shared and group-specific spatial modules analyses based on the shared connectivity loading matrix. **(D)** Temporal brain state analysis at different frequency bands.

]Material description of the envelope predominantly model As seen in Figure 1, multi-subject multi-frequency dFNC tensors of HCs and SZs are constructed using the filter-banked connectivity approach after extracting the ICs of interest by group ICA. The dFNC tensors are then jointly broken down into various factor matrices using the proposed CCPD method under sparsity and low-rank constraints, which are respectively expressed as shared connectivity loading matrix, group-specific time and frequency weights, and group-specific subject intensities.

### 2.1 fMRI data

This paper utilizes the public COBRE fMRI dataset including 145 subjects which is based on a program funded by the National Institutes of Health (NIH) to support outstanding scientists and research institutions in the field of biomedical research. The COBRE dataset provides original fMRI data of 71 SZs and 74 HCs (each group ranging in age from 18 to 65 years old). All subjects do not have a history of neurological disease, intellectual disability, severe head trauma (loss of consciousness for more than 5 min), and drug abuse or dependence in the past 12 months. Diagnostic information was collected using the Structured Clinical Interview for DSM Disorders (SCID). All participants were scanned using a Siemens TIM3.0-Tesla scanner. A multi-echo MPRAGE (MEMPR) sequence was used with the following parameters: TR/TE/TI = 2,530/[1.64, 3.5, 5.36, 7.22, 9.08]/900 ms, flip angle = 7°, FOV = 256 × 256 mm, slice thickness = 176 mm, matrix = 256 × 256 × 176, voxel size = 1 × 1 × 1 mm^3^, number of echoes = 5, pixel bandwidth = 650 Hz, and total scan time = 6 min. Using 5 echoes, the TR, TI, and encoding partition time of MEMPR were similar to those of conventional MPRAGE, resulting in similar GM/WM/CSF contrasts. Resting-state fMRI data acquisition used EPI imaging, with the mutual measurement line (AC-PC) as a reference, and slope sampling correction (TR: 2 s, TE: 29 ms, matrix size: 64 × 64, 32 slices, voxel size: 3.75 × 3.75 × 4.55 mm^3^). During the acquisition process, all subjects were instructed to open their eyes and stare at the fixed cross. The data acquisition lasted 5 min and the number of scans was 146.

After data acquisition, the Statistical Parametric Mapping (SPM) software was used to preprocess the acquired data, including slice timing correction, head motion correction, spatial normalization to the standard Montreal Neurological Institute (MNI) template, and spatial smoothing using a Gaussian kernel with a full width at half maximum (FWHM) of 10 × 10 × 10 mm^3^ to increase the signal-to-noise ratio of fMRI data and to facilitate the alignment of functional data across subjects of the original data (Calhoun et al., 2012).

### 2.2 Multi-subject multi-frequency dFNC tensor obtained by group ICA

We first utilize group ICA with high model order 100 to separate the 145-subject resting-state fMRI data into subject-specific spatial map and time course information for dFNC analysis. Specifically, group ICA is implemented by the GIFT toolbox (https://trendscenter.org/software/). The model orders of first-stage PCA and second-stage PCA are 120 and 100, to avoid expensive memory requirements and retain the important information. Infomax algorithm is chosen, 20 runs are repeated for ICA, and the best run results are selected using ICASSO. The subject-specific spatial maps and time courses are extracted by group ICA. Subsequently, *C* = 41 interesting ICs are chosen using the spatial references which are constructed by multi-scale fMRI Neuromark template (https://trendscenter.org/data/). These 41 interesting ICs can be further divided into seven IC networks (ICNs): subcortical (SUB, 8 ICs), auditory (AUD, 2 ICs), somatomotor (SM, 9 ICs), visual (VIS, 6 ICs), cognitive control (CC, 9 ICs), default mode network (DMN, 5 ICs), and cerebellar (CB, 2 ICs).

The following post-processing methods are performed on the subject-specific time courses of selected 41 ICs: detrending linear, quadratic, and cubic trends, despiking, and band-pass filtering between 0.01 ~ 0.15 Hz using a 5th order Butterworth filter. Subsequently, the filter-banked connectivity method is performed on the processed time courses. Suppose two different time courses *m*(*t*) and *n*(*t*), 2Δ+1 is the window size, μ_*m*_(*t*) and μ_*n*_(*t*) are the windowed sample mean values of *m*(*t*) and *n*(*t*); σ_*m*_(*t*) and σ_*n*_(*t*) are windowed standard deviation values of *m*(*t*) and *n*(*t*), which are defined as follows:


$$\begin{array}{l} \mu_{m}\left(t\right)=\frac{1}{2\Delta +1}\overset{t+\Delta }{\underset{i=t-\Delta }{\sum }}m\left(i\right), \end{array}$$ (1)

$$\begin{array}{l} \mu_{n}\left(t\right)=\frac{1}{2\Delta +1}\overset{t+\Delta }{\underset{i=t-\Delta }{\sum }}n\left(i\right), \end{array}$$ (2)

$$\begin{array}{l} \sigma_{m}\left(t\right)=\sqrt{\overset{t+\Delta }{\underset{i=t-\Delta }{\sum }}{\left(m\left(i\right)-\mu_{m}\left(t\right)\right)}^{2},} \end{array}$$ (3)

$$\begin{array}{l} \sigma_{n}\left(t\right)=\sqrt{\overset{t+\Delta }{\underset{i=t-\Delta }{\sum }}{\left(n\left(i\right)-\mu_{n}\left(t\right)\right)}^{2}.} \end{array}$$ (4)

By convolving a rectangle window with a size of 2Δ+1 = 30 points, the sliding-windowed Pearson correlation *R*_*mn*_(*t*) of time point *t* is defined as:


$$\begin{array}{l} R_{m,n}\left(t\right)=\overset{t+\Delta }{\underset{i=t-\Delta }{\sum }}\frac{\left[m\left(i\right)-\mu_{m}\left(i\right)\right]\left[n\left(i\right)-\mu_{n}\left(i\right)\right]}{\sigma_{m}\left(i\right)\sigma_{n}\left(i\right)}. \end{array}$$ (5)

Since the number of time points is 146, the number of windows is 116, and t of *R*_*mn*_(*t*) ranges from 1 to 116. As *C* = 41, there are *C*(*C*−1)/2 = 820 combinations of sliding-windowed Pearson correlation values for each time point *t*. The sliding-window correlation and its associated definitions are given in Equations 1–5. Along this line, according to the frequency distribution of actual data, we filter the *R*_*mn*_(*t*) with different 3 frequency bands using the infinite impulse response (IIR) filter. According to the frequency spectrum distribution of subject-specific time courses (see Supplementary material), the specific frequencies of these three filter bands are selected as: 0.000–0.025 Hz of band 1; 0.025–0.050 Hz of band 2; 0.100–0.130 Hz of band 3. As analyzed in Section S3 of Supplementary material and displayed in Supplementary Figure S3, the intermediate range (0.05–0.1 Hz) exhibited very tiny signal power and showed no preliminary differences between groups. Therefore, it was excluded from the main analysis to focus on the most informative and potentially disease-relevant frequency components. Finally, a multi-frequency dFNC tensor with a size of 820 × 116 × 3 is generated for each subject. We temporally concatenate the windows and frequency bands and transform the subject-specific multi-frequency dFNC tensor into a matrix with a size of 820 × 348. Considering the subject dimension, the multi-subject multi-frequency dFNC tensor of HCs and SZs can be formed as $X_{\text{H}{\text{C}}_{s}}^{\left(n\right)}\in {\mathbb{R}}^{820\times 348\times 74}$ and $X_{\text{S}{\text{Z}}_{s}}^{\left(n\right)}\in {\mathbb{R}}^{820\times 348\times 71}$ respectively.

### 2.3 The proposed CCPD method under sparsity and low-rank constraints

After obtaining the multi-subject multi-frequency dFNC tensors, we assume that all subjects share the same connectivity but own different temporal, frequency, and subject information for different groups, as the inter-subject temporal difference is larger than the inter-subject connectivity difference. As such, assume *N* tensors 𝓍^(*n*)^(*n* = 1, …, *N*), the number of connectivity combinations is *I*, the number of temporal windows is *J*, the number of frequency bands is *F*, and the number of subjects of group *n* is *K*_*n*_. The proposed CCPD method under sparsity and low-rank constraint is defined as follows:


$$\begin{array}{l} X^{\left(n\right)}=\overset{R}{\underset{r=1}{\sum }}{\text{a}}_{r}•{\text{b}}_{r}^{\left(n\right)}•{\text{c}}_{r}^{\left(n\right)}+\varepsilon^{\left(n\right)},\;n=1,\ldots ,N, \end{array}$$ (6)

where *R* is the number of components, operation “°” is the outer product, $\text{A}=\left\{{\text{a}}_{r}\right\}\in {\mathbb{R}}^{I\times R}$ is the shared connectivity loading matrix, ${\text{B}}^{\left(n\right)}=\left\{{\text{b}}_{r}^{\left(n\right)}\right\}\in {\mathbb{R}}^{JF\times R}$ is the group-specific time and frequency weights, ${\text{C}}^{\left(n\right)}=\left\{{\text{c}}_{r}^{\left(n\right)}\right\}\in {\mathbb{R}}^{K_{n}\times R}$ is the group-specific subject intensities, and **ε**^(*n*)^ is the residual. Equation 6 defines the CCPD decomposition of the dFNC tensor.

To emphasize the significant connectivity, the L1-norm sparsity constraint on the connectivity loading matrix is imposed and the low-rank approximation is added on ${\text{b}}_{r}^{\left(n\right)}$, respectively. Therefore, the objective function of the proposed constrained CCPD method is


$$\begin{array}{l} \underset{{\text{a}}_{r},{\text{b}}_{r}^{\left(n\right)},{\text{c}}_{r}^{\left(n\right)}}{\min}\overset{N}{\underset{n=1}{\sum }}||{\text{a}}_{r}•{\text{b}}_{r}^{\left(n\right)}•{\text{c}}_{r}^{\left(n\right)}|{|}_{F}+\lambda ||\text{A}|{|}_{1}+\gamma \text{rank}\left({\text{B}}^{\left(n\right)}\right), \end{array}$$ (7)

where “||**·**||_*F*_” and “||**·**||_1_” denote Frobenius norm and L1-norm, function “rank(·)” computes the rank of the given matrix, λ and γ are constant which control the weight of penalty terms.

Define matrix **Da**∈ℂ^*R*×*R*^:


$$\begin{array}{l} \text{D}\text{a}=\overset{N}{\underset{n=1}{\sum }}\left[\begin{array}{ccc} {\text{c}}_{1}^{\left(n\right)\text{T}}{\text{X}}^{\left(1\cdot \cdot ,n\right)\text{T}}{\text{b}}_{1}^{\left(n\right)} & \cdots & {\text{c}}_{1}^{\left(n\right)\text{T}}{\text{X}}^{\left(I\cdot \cdot ,n\right)\text{T}}{\text{b}}_{1}^{\left(n\right)}\\ \vdots & \ddots & \vdots \\ {\text{c}}_{R}^{\left(n\right)\text{T}}{\text{X}}^{\left(1\cdot \cdot ,n\right)\text{T}}{\text{b}}_{R}^{\left(n\right)} & \cdots & {\text{c}}_{R}^{\left(n\right)\text{T}}{\text{X}}^{\left(I\cdot \cdot ,n\right)\text{T}}{\text{b}}_{R}^{\left(n\right)} \end{array}\right], \end{array}$$ (8)

by exploiting alternating least squares (ALS), the shared connectivity loading matrix **A** with L1-norm sparsity constraint is updated based on minimization (Equation 8):


$$\{\begin{array}{l} A^{\text{T}}={\left(\sum_{n=1}^{N}\left(B^{(n)\text{T}}B^{\left(n\right)}\right)*(C^{(n)\text{T}}C^{\left(n\right)})\right)}^{-1}(Da-\lambda )\\ a_{r}\leftarrow \frac{a_{r}}{{||a_{r}||}_{F}},\;r\in \{1,\ldots ,R\}\cdot \end{array}$$ (9)

where ${\text{X}}^{\left(1\ldots n\right)}\in {\mathbb{R}}^{J_{n}\times K_{n}}$ is the ith slice matrix of 𝓍^(*n*)^, operation “*” is the Hardmard product. Equation 9 updates the shared connectivity loading matrix under the L1-norm sparsity constraint. The least-squares fit of group-specific time and frequency weights matrices **B**^(*n*)^(*n* = 1, …, *N*) under low-rank approximation can be presented as follows:


$$\begin{array}{l} {\text{B}}^{\left(n\right)}\leftarrow \underset{{\text{B}}^{\left(n\right)}}{\min}\frac{1}{2}||{\text{X}}_{\left(2\right)}^{\left(n\right)}{\left({\text{C}}^{\left(n\right)}⊙\text{A}\right)}^{†}-{\text{B}}^{\left(n\right)}|{|}_{F}^{2}+\gamma \mathrm{rank}\left({\text{B}}^{\left(n\right)}\right), \end{array}$$ (10)

where ${\text{X}}_{\left(2\right)}^{\left(n\right)}$ is the mode-2 unfolding matrix of 𝓍^(*n*)^ and operation “⊙” is the Khatri-Rao product.

Let $\text{Z}={\text{X}}_{\left(2\right)}^{\left(n\right)}{\left(\text{C}⊙{\text{A}}^{\left(n\right)}\right)}^{†}$, as the rank(**B**^(*n*)^) is a non-convex term, this paper exploits the nuclear norm of **B**^(*n*)^ denoted as $||{\text{B}}^{\left(n\right)}|{|}_{*}$ to approximate the low-rank of **B**^(*n*)^, and thus Equation 10 can be transformed as:


$$\begin{array}{l} {\text{B}}^{\left(n\right)}\leftarrow \underset{{\text{B}}^{\left(n\right)}}{\min}\frac{1}{2}||{\text{Z}}^{\left(n\right)}-{\text{B}}^{\left(n\right)}|{|}_{F}^{2}+\gamma ||{\text{B}}^{\left(n\right)}|{|}_{*}, \end{array}$$ (11)

Equation 11 can be solved by introducing a relaxing matrix, and thus Equation 11 becomes


$$\begin{array}{l} {\text{B}}^{\left(n\right)}\leftarrow \underset{{\text{B}}^{\left(n\right)}}{\min}\frac{1}{2}||{\text{Z}}^{\left(n\right)}-{\text{B}}^{\left(n\right)}|{|}_{F}^{2}+\gamma ||{\text{Q}}^{\left(n\right)}|{|}_{*}\text{, s.t.}{\text{Q}}^{\left(n\right)}={\text{B}}^{\left(n\right)}. \end{array}$$ (12)

By exploiting the Lagrangian method, Equation 12 can be solved by minimizing the objective function:


$$\begin{array}{l} L=\frac{1}{2}||Z^{\left(n\right)}-B^{\left(n\right)}|{|}_{F}^{2}+\gamma ||Q^{\left(n\right)}|{|}_{*}+\langle Y^{\left(n\right)},Q^{\left(n\right)}-B^{\left(n\right)}\rangle \\ \;+\frac{\sigma^{\left(n\right)}}{2}||Q^{\left(n\right)}-B^{\left(n\right)}|{|}_{F}^{2}, \end{array}$$ (13)

where ${\text{Y}}^{\left(n\right)}\in {\mathbb{R}}^{J_{n}\times R}$ is a Lagrangian multiplier, σ^(*n*)^ is a hyper-parameter, and “ < .,. >” denotes the inner product. The update of **Q**^(*n*)^ can be derived as follows:


$$\begin{array}{l} {\text{Q}}^{\left(n\right)}\leftarrow \underset{{\text{Q}}^{\left(n\right)}}{\min}\frac{\gamma }{\sigma^{\left(n\right)}}||{\text{Q}}^{\left(n\right)}|{|}_{*}+\frac{1}{2}||{\text{Q}}^{\left(n\right)}-\left({\text{B}}^{\left(n\right)}-\frac{{\text{Y}}^{\left(n\right)}}{\sigma^{\left(n\right)}}\right)|{|}_{F}^{2}. \end{array}$$ (14)

This can be solved by the tensor robust PCA method with a tensor nuclear norm (Lu et al., 2019). The update of **B**^(*n*)^ is


$$\begin{array}{l} {\text{B}}^{\left(n\right)}\leftarrow \frac{1}{1+\sigma^{\left(n\right)}}\left(\frac{{\text{Q}}^{\left(n\right)}}{\sigma^{\left(n\right)}}+{\text{Y}}^{\left(n\right)}+{\text{Z}}^{\left(n\right)}\right), \end{array}$$ (15)

and the matrix **Y**^(*n*)^ and parameter σ^(*n*)^(*n* = 1, …, *N*) can be updated as follows:


$$\begin{array}{l} \begin{array}{cc} {\text{Y}}^{\left(n\right)} & \leftarrow {\text{Y}}^{\left(n\right)}+\sigma^{\left(n\right)}\left({\text{Q}}^{\left(n\right)}-{\text{B}}^{\left(n\right)}\right)\\ \sigma^{\left(n\right)} & \leftarrow \min\left(\varphi \sigma^{\left(n\right)},\sigma_{\max}\right) \end{array}, \end{array}$$ (16)

where φ and σ_max_ are constant values.

Define matrix ${\text{D}}_{{\text{C}}^{\left(n\right)}}\in {\mathbb{C}}^{R\times R}:$


$$\begin{array}{l} {\text{D}}_{{\text{C}}^{\left(n\right)}}=\left[\begin{array}{ccc} {\text{B}}_{1}^{\left(n\right)\text{T}}{\text{X}}^{\left(\cdot 1\cdot ,n\right)\text{T}}{\text{a}}_{1} & \cdots & {\text{b}}_{1}^{\left(n\right)\text{T}}{\text{X}}^{\left(\cdot J_{n}\cdot ,n\right)\text{T}}{\text{a}}_{1}\\ \vdots & \ddots & \vdots \\ {\text{b}}_{R}^{\left(n\right)\text{T}}{\text{X}}^{\left(\cdot 1\cdot ,n\right)\text{T}}{\text{a}}_{R} & \cdots & {\text{b}}_{R}^{\left(n\right)\text{T}}{\text{X}}^{\left(\cdot J_{n}\cdot ,n\right)\text{T}}{\text{a}}_{R} \end{array}\right] \end{array}$$ (17)

the update of group-specific subject intensities **C**^(*n*)^(*n* = 1, …, *N*) is based on the least-squares fit of Equation 7:


$$\{\begin{array}{l} C^{(n)\text{T}}={\left(\sum_{n=1}^{N}\left(A^{\text{T}}A\right)*(B^{(n)\text{T}}B^{\left(n\right)})\right)}^{-1}D_{C^{\left(n\right)}}\\ c_{r}^{\left(n\right)}\leftarrow \frac{c_{r}^{\left(n\right)}}{{||c_{r}^{\left(n\right)}||}_{F}},r=1,\ldots ,R,n=1,\ldots ,N \end{array}$$ (18)

where ${\text{X}}^{\left(\cdot j\cdot ,n\right)}\in {\mathbb{R}}^{I\times K_{n}}$ is the ith fiber matrix of 𝓍^(*n*)^. Equations 12–18 describe the low-rank approximation procedure and the updates for B(n) and C(n). The loading matrices **A**, **B**^(*n*)^, and **C**^(*n*)^(*n* = 1, …, *N*) are alternatively updated until convergence. The detailed procedure of the proposed CCPD method under sparsity and low-rank constraints (shorted as SLRCCPD) is illustrated in Table 1.

**Table 1:** Implementation of the proposed CCPD method under sparsity and low-rank constraints.

Input: multi-group multi-frequency dFNC tensor X^(*n*)^ (*n* = 1, …, *N*), the number of groups *N*, the number of components *R*, the minimum of iteration error ε_itermin_, and the maximum number of iterations iter_max_.
Randomly initialize **A**, **B**^(*n*)^, and **C**^(*n*)^, iter1 = 0, σ = 10^−6^, $\sigma_{\text{max}}=10^{6}$, φ = 1.1, and calculate the initial error ε_0_ based on (7).
**While** ε_iter_>ε_iter_min_ or iter < iter_max_ **do**
iter1 = iter1+1;
First update **A** based on (9);
iter2 = 0;
**For** (iter2 = 0; iter2 < 1000; iter2++) **do**
Update **Q**^(*n*)^ by (14);
Update **B**^(*n*)^ using (15);
Update matrix **Y**^(*n*)^ and parameter σ^(*n*)^ using (16);
**End for**
Update **C**^(*n*)^ (*n* = 1, …, *N*) using (18);
Calculate the error ε_iter_ based on (7);
**End while**
**Output:** **A**, **B**^(*n*)^, and **C**^(*n*)^ (*n* = 1, …, *N*).

### 2.4 Shared dynamic connectivity modules analysis

To determine the optimal number of dynamic connectivity modules, we systematically evaluated *R* values ranging from 3 to 7 using SLRCCPD. For each *R*, we assessed reconstruction error, goodness–of–fit, module pair discriminability, and clustering stability. After extracting the shared dynamic connectivity loading matrix **A**∈ℝ^820 × 5^ using the proposed method with parameters *I* = 820, *R* = 5, λ = 0.05, and γ = 20, the symmetric shared dynamic connectivity matrix ${\text{A}}_{r}\in {\mathbb{R}}^{41\times 41}$ of each module can be acquired by transforming each vector **a**_*r*_ of **A**. First, the within-ICN connectivity strength of each threshold ICN *Ai*_*r*_(*icn*_1_) is calculated to evaluate the connectivity strength among ICs in a certain ICN (*icn*_1_):


$$\begin{array}{l} Ai_{r}\left(icn_{1}\right)=\frac{1}{2M}\underset{s_{1},s_{2}\in m}{\sum }{\text{A}}_{r}\left(s_{1},s_{2}\right), \end{array}$$ (19)

where **A**_*r*_(*s*_1_, *s*_2_) is the element of shared dynamic connectivity matrix **A**_*r*_, *s*_1_ and *s*_2_ are the *s*_1_ th and *s*_2_ th ICs(1 ≤ *s*_1_, *s*_2_ ≤ *S* and *s*_1_≠*s*_2_), respectively; *m* and *M*(*M*>1) respectively are the ICs set and the number of ICs of ICN (*icn*_1_). Second, the between-ICN connectivity strength *Ab*_τ_(*icn*_1_, *icn*_2_) between two ICNs *icn*_1_ and *icn*_2_ is computed to evaluate the between-ICN connectivity strength between two ICNs:


$$\begin{array}{l} Ab_{r}\left(icn_{1},icn_{2}\right)=\frac{1}{MN}\underset{s_{1}\in m,s_{2}\in n}{\sum }{\text{A}}_{r}\left(s_{1},s_{2}\right), \end{array}$$ (20)

where *m* and *M*(*M*>1) respectively are the ICs set and the number of ICs of ICN *icn*_1_, and *n* and *N*(*N*>1) respectively are the ICs set and the number of ICs of ICN *icn*_2_.

To comprehensively measure the within-ICN and between-ICN connectivity of ICNs and find the ICN with the highest contribution of overall connectivity strength, a connectivity contribution measure is calculated as


$$\begin{array}{l} p_{r}\left(icn_{1}\right)=Ai_{r}\left(icn_{1}\right)+\underset{\begin{array}{c} icn_{1}\in l\\ icn_{1}\neq icn_{2} \end{array}}{\sum }\frac{1}{L-1}Ab_{r}\left(icn_{1},icn_{2}\right), \end{array}$$ (21)

where *l* and *L* are the ICNs set of the number of ICNs of the rth module, respectively. Equation 21 defines the connectivity contribution measure for each module. To statistically assess group differences in module contributions, we performed Mann-Whitney U tests on the subject intensity matrix ${\text{C}}_{HC}^{\left(f\right)}$ and ${\text{C}}_{SZ}^{\left(f\right)}$. For each module, we compared the HC group and SZ group. Effect sizes were quantified using the rank biserial correlation.

### 2.5 Group-specific dynamic connectivity modules analysis

The group-specific dynamic connectivity modules are further estimated to analyze the group difference between HCs and SZs:


$$\{\begin{array}{l} A_{\text{HC}}=X_{\left(3\right)}^{\left(1\right)}{\left(B^{\left(1\right)}⊙C^{\left(1\right)}\right)}^{†\text{T}}\\ A_{\text{SZ}}=X_{\left(3\right)}^{\left(2\right)}{\left(B^{\left(2\right)}⊙C^{\left(2\right)}\right)}^{†\text{T}} \end{array}$$ (22)

where operation “⊙” is the Khatri-Rao product, ${\text{A}}_{\text{HC}}\in {\mathbb{R}}^{I\times R}$ and ${\text{A}}_{\text{SZ}}\in {\mathbb{R}}^{I\times R}$ are the dynamic connectivity modules of HCs and SZs, respectively. ${\text{X}}_{\left(3\right)}^{\left(1\right)}\in {\mathbb{R}}^{I\times J_{1}K_{1}}$ and ${\text{X}}_{\left(3\right)}^{\left(2\right)}\in {\mathbb{R}}^{I\times J_{2}K_{2}}$ are respectively the mode-3 unfolding matrix of the multi-subject dFNC tensors for HCs and SZs. The between-ICN connectivity strength matrices *Ab*_*r*_(*icn*_1_, *icn*_2_) of HCs and SZs are calculated to investigate the group connectivity difference between HCs and SZs.

### 2.6 Temporal brain state analysis at different filter bands

Based on the extracted time and frequency weights matrices of HCs **B**^(1)^∈ℝ^348 × 5^ and SZs **B**^(2)^∈ℝ^348 × 5^, the extracted shared time-varying weights under three frequency bands *f* (*f* = 1, 2, 3) can be obtained by dividing **B**^(1)^ and **B**^(2)^ evenly into three parts **B**^(1, *f*)^∈ℝ^116 × 5^ and **B**^(2, *f*)^∈ℝ^116 × 5^ (*f* = 1, 2, 3). The subject-specific time-varying weights ${\text{H}}_{\text{HC}}^{\left(r,f\right)}\in {\mathbb{R}}^{116\times 74}$ and ${\text{H}}_{\text{SZ}}^{\left(r,f\right)}\in {\mathbb{R}}^{116\times 71}$ (*r* = 1, …, 5, *f* = 1, 2, 3) of dynamic module *r* and frequency band *f* are obtained by multiplying the column vectors of ${\text{B}}^{\left(n,f\right)}\left({\text{b}}_{r}^{\left(n,f\right)},n=1,2\right):$ with the corresponding column vectors of subject intensities ${\text{C}}^{\left(n\right)}\left({\text{c}}_{r}^{\left(n\right)},n=1,2\right):$


$$\{\begin{array}{l} H_{\text{HC}}^{\left(r,f\right)}=b_{r}^{\left(1,f\right)}*{\left(c_{r}^{\left(1\right)}\right)}^{\text{T}}\\ H_{\text{SZ}}^{\left(r,f\right)}=b_{r}^{\left(2,f\right)}*{\left(c_{r}^{\left(2\right)}\right)}^{\text{T}} \end{array}$$ (23)

The ${\text{H}}_{\text{HC}}^{\left(r,f\right)}$ and ${\text{H}}_{\text{SZ}}^{\left(r,f\right)}$ of all 5 dynamic models are further temporally concatenated as ${\text{H}}_{\text{HC}}^{\left(f\right)}\in {\mathbb{R}}^{116\times 370}$ and ${\text{H}}_{\text{SZ}}^{\left(f\right)}\in {\mathbb{R}}^{116\times 355}$. As defined in Equation 23, the subject-specific time-frequency weights are obtained as follows. The popular k-means clustering method is respectively applied to ${\text{H}}_{\text{HC}}^{\left(f\right)}$ and ${\text{H}}_{\text{SZ}}^{\left(f\right)}$. We use the elbow criterion for k-means clustering to select the ideal state number, and obtain the number of states is 6. We remove clusters with less than 3% of samples after clustering, and the final state number is 3. After clustering, to clearly identify the clustering effect and group differences, the nonparametric Mann–Whitney *U*-test is performed on the HCs and the SZs to compare the group differences at different time states. Finally, we utilize the following three measure parameters to compare the time-varying weight differences of difference groups in different frequency bands: (1) DT: the average duration of the time spent in a state before transitioning to the next state; (2) FT: the percentage of time spent in a state; (3) NT: the number of transitions switched from one temporal state to another state for a subject.

## 3 Results

### 3.1 ICNs connections results of different shared connectivity matrices

**Figure 2:**
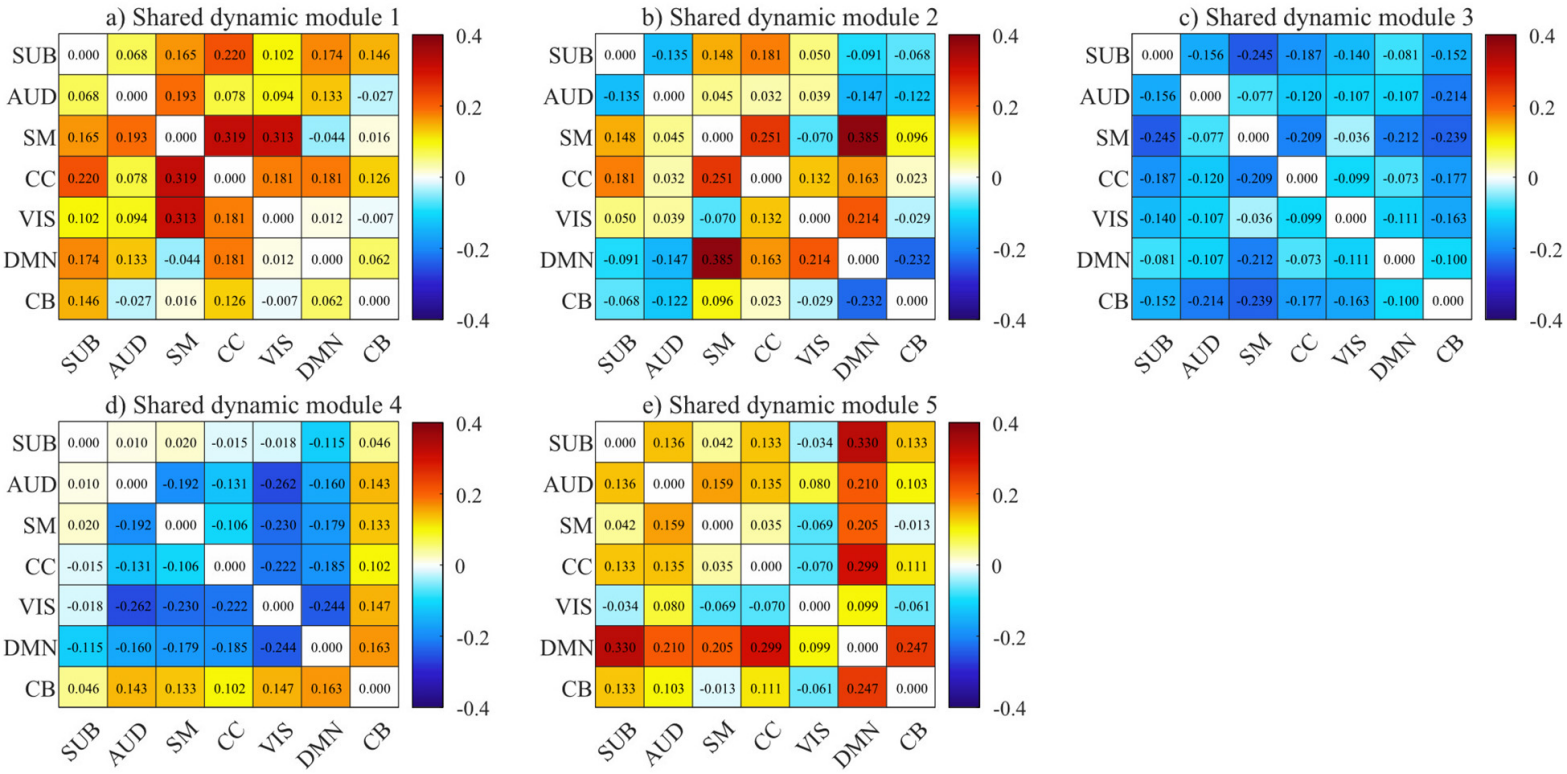
The between-ICN connectivity strength *Ab*_*r*_(*icn*_1_, *icn*_2_) linkages between all 7 ICNs of five different shared dynamic modules estimated by the proposed SLRCCPD. The shared dynamic modules are shown in **(a–e)**.

It is possible to assess the relationships within and between ICNs in the shared dynamic module since **A** depicts the shared dynamic topological connectivity of the five modules. According to Equations 19 and 20, the within-ICN connectivity strength *Ai*_*r*_(*icn*_1_) of a threshold ICN *icn*_1_ and between-ICN connectivity strength *Ab*_*r*_(*icn*_1_, *icn*_2_) between two ICNs *icn*_1_ and *icn*_2_ can be calculated using the symmetric shared dynamic connectivity matrix **A**_*r*_ of shared dynamic module *r*. Firstly, Figure 2 shows between-ICN connectivity strength *Ab*_*r*_(*icn*_1_, *icn*_2_) among SUB, AUD, SM, CC, VIS, DMN, and CB in these five shared dynamic modules. It's important to note that the normalized between-ICN connectivity strength *Ab*_*r*_(*icn*_1_, *icn*_2_) values between ICNs vary depending on different shared dynamic modules, suggesting that each module has distinct functional connectivity characteristics. The majority of the connections in shared dynamic module 1 exhibit positive *Ab*_*r*_(*icn*_1_, *icn*_2_) values, as shown in Figure 2a. In particular, SM exhibits comprehensively strong positive *Ab*_*r*_(*icn*_1_, *icn*_2_) values (i.e., *Ab*_*r*_(*icn*_1_, *icn*_2_) >0.315) with CC and VIS, and CC also exhibits substantial positive connectivity with SUB, VIS, and DMN. For shared dynamic module 2, SM exhibits high positive *Ab*_*r*_(*icn*_1_, *icn*_2_) values not only with DMN but also with CC. CC presents positive connections with other ICNs in both shared dynamic modules 1 and 2. DMN shows positive *Ab*_*r*_(*icn*_1_, *icn*_2_) values with SM, CC, and VIS, while displays negative *Ab*_*r*_(*icn*_1_, *icn*_2_) values with CB, AUD, and SUB. DMN shows positive *Ab*_*r*_(*icn*_1_, *icn*_2_) values with SM, CC, and VIS, while displays negative *Ab*_*r*_(*icn*_1_, *icn*_2_) values with CB, AUD, and SUB. All between-ICN connections in shared dynamic module 3 and most of the between-ICN connections in shared dynamic module 4 are negative, with the exception of the between-ICN connections associated with CB and SUB exhibiting week positive *Ab*_*r*_(*icn*_1_, *icn*_2_) in shared dynamic module 4 values with AUD and SM. VIS acquires higher negative connections with AUD, SM, CC, and DMN. Strong positive connections between DMN and other ICNs are demonstrated in shared dynamic module 5, particularly for the *Ab*_*r*_(*icn*_1_, *icn*_2_) value between SUB and CC.

### 3.2 Connectivity contribution analysis of ICNs

**Figure 3:**
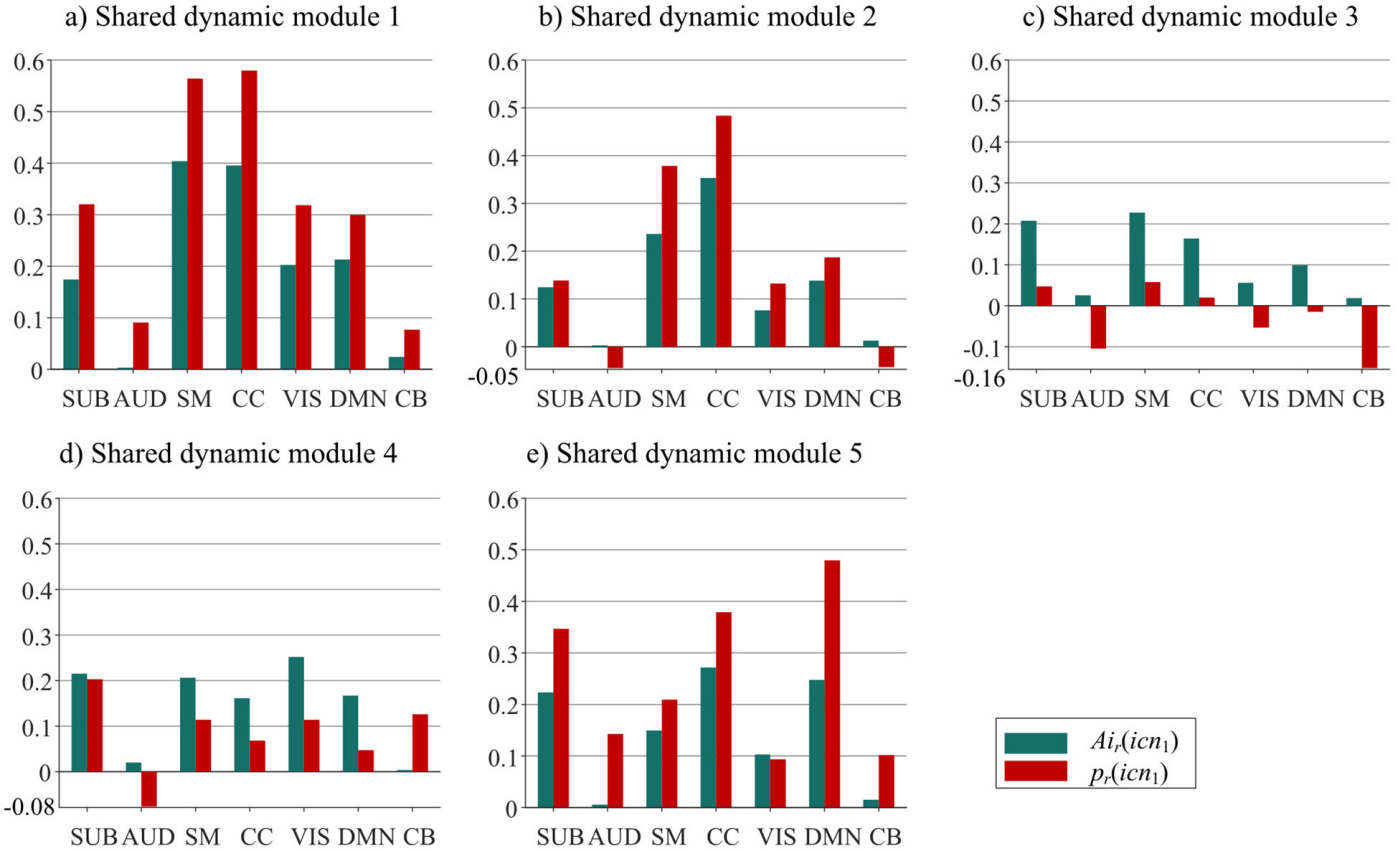
The within-ICN connectivity strength *Ai*_*r*_(*icn*_1_) (green) and connectivity contribution measure *p*_*r*_(*icn*_1_) (red) for all 7 ICNs in five shared dynamic module **(a–e)**.

To evaluate the contribution degree of different ICNs in each shared dynamic module, Figure 3 further insights into the within-ICN connectivity strength *Ai*_*r*_(*icn*_1_) and connectivity contribution measure *p*_*r*_(*icn*_1_) of ICNs in each module. Figure 3 shows that SM and CC have the greatest positive *Ai*_*r*_(*icn*_1_) values and *p*_*r*_(*icn*_1_) values in shared dynamic modules 1 and 2. This indicates the connectivity contribution of SM and CC are significant in shared dynamic modules 1 and 2. AUD and CB exhibit very low *Ai*_*r*_(*icn*_1_) values in all 5 dynamic modules, suggesting that the ICs within AUD and CB have large positive and negative connection variations. Moreover, AUD has negative *p*_*r*_(*icn*_1_) values in shared dynamic modules 2, 3, and 4, while CB has negative *p*_*r*_(*icn*_1_) values in shared dynamic modules 2 and 3. This indicates the negative contribution of AUD and CB. The *Ai*_*r*_(*icn*_1_) values of all ICNs are larger than the corresponding *p*_*r*_(*icn*_1_) values for dynamic module 1, owing to the majority of positive *Ab*_*r*_(*icn*_1_, *icn*_2_) values in module 1 as shown in Figures 2a, 3a. However, the *Ai*_*r*_(*icn*_1_) values of all ICNs are smaller than the corresponding *p*_*r*_(*icn*_1_) values for shared dynamic module 4, and thus CB shows positive connections with other ICNs in dynamic module 4. In shared dynamic module 5, DMN shows the positive *Ab*_*r*_(*icn*_1_, *icn*_2_) value and the highest *p*_*r*_(*icn*_1_) value, and results in the highest *Ab*_*r*_(*icn*_1_, *icn*_2_) values with other ICNs as shown in Figure 2e.

### 3.3 Group-specific dynamic modules of HCs and SZs

**Figure 4:**
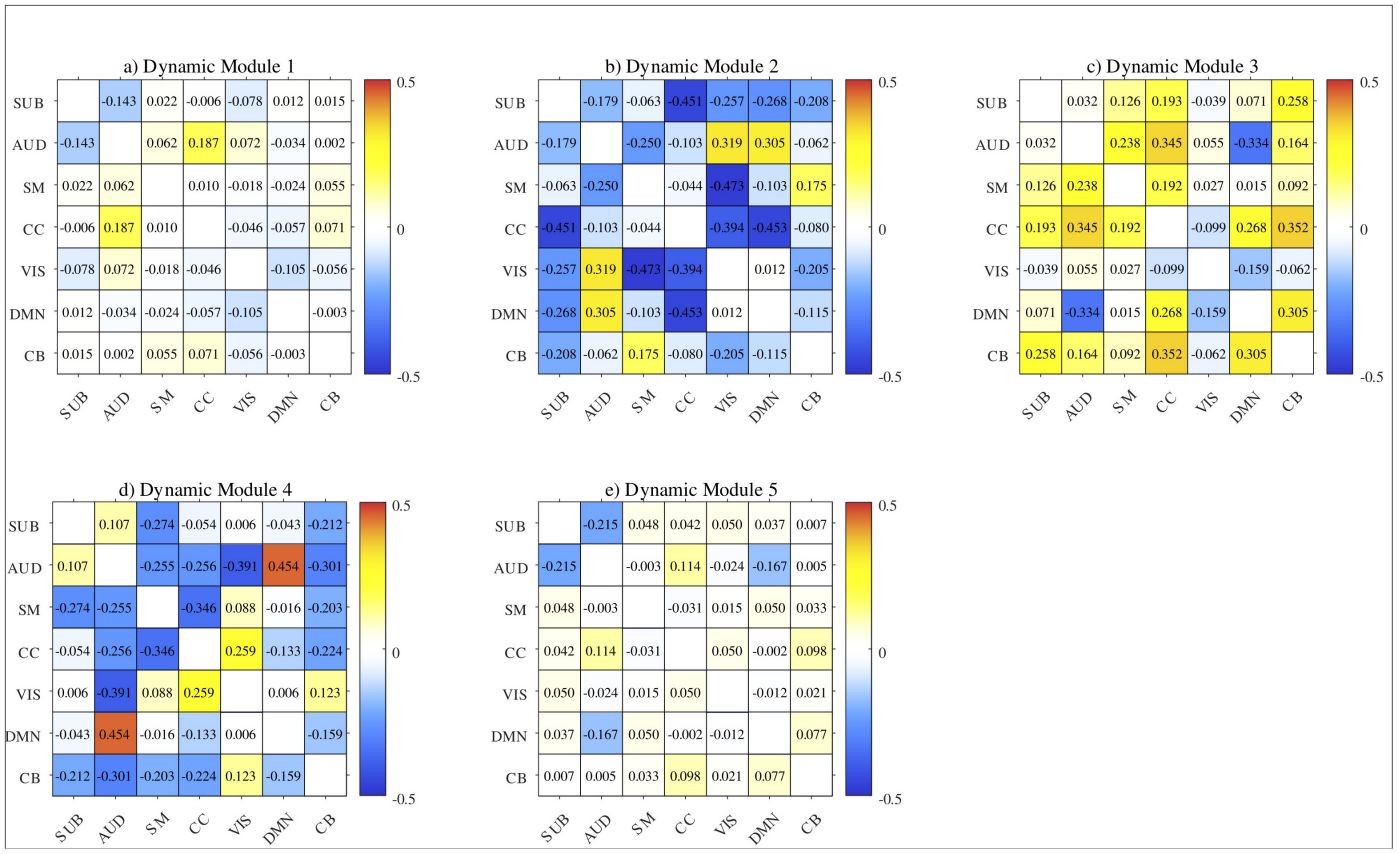
Difference matrices between HCs and SZs of between ICN connectivity strength *Ab*_*r*_(*icn*_1_, *icn*_2_) for the five dynamic modules. Dynamic modules 1–5 are shown in **(a–e)**, respectively.

The group-specific dynamic modules of HCs and SZs are computed according to Equation 22. Figure 4 displays the difference matrices between HCs and SZs of between-ICN connectivity strength linkages for five group-specific dynamic modules. The group-specific dynamic modules 1 and 5 of HCs and SZs exhibit low difference of topographical connection linkages, especially for connections with higher *Ab*_*r*_(*icn*_1_, *icn*_2_) values. The connectivity strength values between DMN and SUB, SM and CC, SM and AUD, and SM and SUB are stronger in HCs than in SZs. For dynamic module 2, most of the between-ICN connections in HCs are lower than those in SZs. The most connections of HCs show higher connectivity strength values than those of SZs in dynamic module 3, but shows lower connectivity strength values in dynamic module 4. The connection difference of DMN and AUD in dynamic module 3 and in dynamic module 4 shows contrary results. In reality, the majority of connections of HCs and SZs have opposite positive and negative signs in these two module. Specifically, in dynamic module 4 of SZs, DMN and AUD have the lowest negative *Ab*_*r*_(*icn*_1_, *icn*_2_) value, and CB obtains positive connections with other ICNs. Most connections among SUB, AUD, SM, and CC are negative in dynamic module 4 of HCs, except for the connection between AUD and SUB. These are contrary to those in dynamic module 3 of SZs and HCs.

To complement the qualitative observations in Figure 4, we performed statistical comparisons on the subject intensity matrix C. The results revealed significant group differences in modules 4 and 5, with module 4 showing lower intensities in HC and module 5 showing higher intensities in HC compared to SZ (see Supplementary Table S2 for detailed results). Modules 1–3 did not reach significance, indicating that group differences in these modules are more related to the sign of individual connections rather than overall module intensity.

### 3.4 Temporal state analysis of different frequency bands

**Figure 5:**
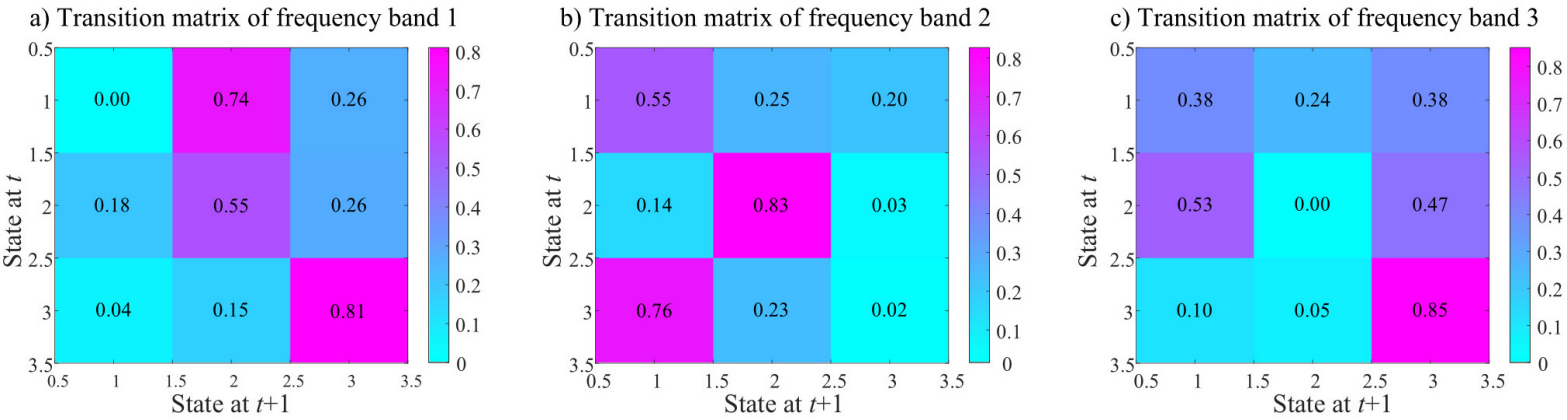
The state transition matrices of the three states after respective clustering **H**^(1)^, **H**^(2)^, and **H**^(3)^ are shown in **(a–c)**, averaged across all subjects (HCs and SZs combined).

The proposed SLRCCPD in this paper imposes a low-rank constraint on the group-specific time and frequency weights **B**^(*n*)^ (*n* = 1, 2) with the objective of enhancing the classification of time and frequency bands and facilitating a more detailed investigation of the shifts and differences in time between different frequency bands. The k-means clustering method is applied to ${\text{H}}^{\left(f\right)}=\left[{\text{H}}_{\text{HC}}^{\left(f\right)},{\text{H}}_{\text{SZ}}^{\left(f\right)}\right]\in {\mathbb{R}}^{116\times 725}$ (*f* = 1, 2, 3) for the purpose of performing different time state analyses for different frequency bands. The optimal number of clusters estimated by the Euclidean distance and elbow criterion is 6. However, after clustering, three states were obtained by removing clusters with sample sizes less than 3%. Figure 5 illustrates the transition probabilities between the 3 states after respectively clustering **H**^(1)^, **H**^(2)^, and **H**^(3)^. In particular, for frequency band 1, the probability of remaining in state 3 and transitioning from state 1 to state 2 is greater than 0.70, but the probability of remaining in state 1 is as low as 0, and the probability of transitioning from state 3 to state 1 is likewise low. For frequency band 2, the probability of remaining in state 2 and transitioning from state 3 state 3 to state 1 is greater than 0.75, while the probability of remaining in state 3 is close to 0. Similar to frequency band 1, frequency band 3 gets the highest probability (0.85) of remaining in state 3, the lowest chance of remaining in state 2 (0), and the probability of transitioning from state 3 to state 2 is close to 0. These results imply that alterations in different frequencies impact the transition probabilities between the three states.

### 3.5 Temporal state differences between HCs and SZs

**Figure 6:**
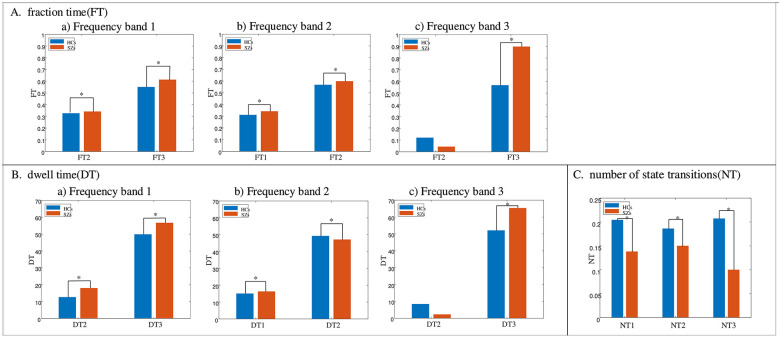
**(A)** Fractional time (FT) values at different states of HCs and SZs after respectively clustering **H**^(1)^, **H**^(2)^, and **H**^(3)^. for frequency bands 1–3 (subplots a–c). **(B)** Dwell time (DT) values similarly for frequency bands 1–3 (subplots a–c). **(C)** Number of state transitions (NT) values (global) after respectively clustering **H**^(1)^, **H**^(2)^, and **H**^(3)^. The significantly different FT and DT values between HCs and SZs by performing Mann-Whitney U-tests are shown. “FT1,” “FT2,” and “FT3” respectively denote the FT values in states 1, 2, and 3; “DT1,” “DT2,” and “DT3” denote the DT values in states 1, 2, and 3; “NT1,” “NT2,” and “NT3,” respectively, denote the NT values in frequency bands 1, 2, and 3.

The DT, FT, and NT measurements of each state for HCs and SZs are calculated separately after respectively clustering **H**^(1)^, **H**^(2)^, and **H**^(3)^ of different frequency bands, and the Mann-Whitney U-test is performed on the DT and FT values of each state between HCs and SZs. The statistically significant differences (*p* < 0.05) between DT and FT values at certain states between HCs and SZs are shown in Figures 6A, B. In general, frequency bands 1 and 3, which contain most of the energy of the time courses, exhibit similar results including the DT and FT values in state 3 of HCs smaller than those of SZs, higher DT and FT values in state 3 than those in state 2, lower FT values in state 2 than frequency band 2, NT values of HCs larger than those of SZs. Figures 6Aa, Ab show that for frequency band 1, states 2 and 3 of HCs had much lower FT and DT values than those of SZs. For the frequency band 2, DT and FT values in state 2 exhibit opposite tendencies between HCs and SZs. Specifically, SZs show significantly higher FT values in states 1 and 2 and DT values in state 1 than HCs, while SZs exhibits significantly lower DT values in state 2 than HCs. Frequency band 3 displays larger FT changes between HCs and SZs (SZs larger than HCs) than frequency bands 1 and 2. Figure 6C depicts the NT values of HCs and SZs over three frequency bands. SZs consistently have lower NT values than HCs in all three frequency bands.

### 3.6 Compared with other methods

In order to examine the effectiveness of the L1-norm sparsity constraint, a two-sample t-test is performed on the **A**, values of the two dynamic modules. Table 2 compares the number of significantly different dynamic module pairs in each dynamic module for CCPD, CCPD under low-rank constraint (LRCCPD), CCPD under L1-norm sparsity constraint (SCCPD), and SLRCCPD proposed in this paper (*p* < 0.05). It can be observed from Table 2 that the SCCPD and SLRCCPD obtained higher number of significantly different dynamic module pairs than CCPD, LRCCPD and SCCPD. This implies that the L1-norm constraint can improve the difference of the dynamic spatial modules.

**Table 2:** Performance comparison of the proposed SLRCCPD against CCPD, LRCCPD, and SCCPD in terms of significant differences detected in dynamic modules (D1-D5) and frequency bands (B1–B3).

Methods	D1	D2	D3	D4	D5	B1	B2	B3
CCPD	1	0	1	0	0	8	6	6
LRCCPD	2	2	2	1	0	8	6	6
SCCPD	3	2	3	4	1	9	6	6
SLRCCPD (proposed)	3	3	4	3	3	10	6	6

Besides, a two-sample t-test on time-varying weights across states in each frequency band is used to assess the effectiveness of the low-rank constraint. As shown in Table 2, all four tensor decomposition methods yield the same number of significant state pairs in frequency bands 2 and 3. However, SLRCCPD identifies more significant state pairs in frequency band 1, which contains the majority of time course energy. This shows that low-rank constraints facilitate the extraction of meaningful states.

## 4 Conclusion and discussion

In this paper, we first construct the multi-subject multi-frequency dFNC tensors of HCs and SZs from the subject-specific time course results extracted by group ICA via the filter-banked connectivity approach and then develop a novel spatiotemporal SLRCCPD framework for jointly separating these tensors into shared connectivity loading matrix, group-specific time and frequency weights and group-specific subject intensity. A fresh viewpoint on the investigation of biomarkers for the identification of schizophrenia is offered by the dynamic spatiotemporal differences that are mined between HCs and SZs at various temporal states and frequency bands. Through the filter-banked connectivity approach, we discover high-frequency state information that typical window-based methods could not record. Significant differences between HCs and SZs in shared dynamic modules are explored, with most HCs and SZs connections showing opposite connectivity values in group-specific dynamic modules 3 and 4. By applying the k-means clustering algorithm to the subject-specific time and frequency weights of the five dynamic models, significantly different temporal states between HCs and SZs in three frequency bands are yielded. Specifically, the FT and DT values of SZs in state 3 at low-frequency band 1 and high-frequency band 3 are significantly higher than those of HCs, while SZs present significantly lower NT values than HCs at all frequency bands. These significant differences in terms of ICN connections and time-frequency state analysis using the proposed method provide a new insight view of understanding functional dynamics and cognition impairment of schizophrenia.

Schizophrenia is a very common brain disease. Since the spatial brain network is not fully involved and the brain structure and function of SZs differ from those of HCs, we applied an L1-norm sparsity constraint to the shared connectivity matrix to extract significant dynamic connections and avoid model overfitting. In addition, the low-rank constraint makes component analysis and grouping easier (Kuang et al., 2024; Calhoun and Adali, 2012). Therefore, to enhance the clustering quality of time states under various frequency bands, we use the low-rank constraint on group-specific time and frequency weights to get low-rank and significant time and frequency weights. The results of Table 2 verify the low-rank efficacy and sparsity of the proposed approach.

The ICNs connection results of shared dynamic connectivity modules as illustrated in Figure 2 show that the majority of ICNs in shared dynamic modules 1, 2, and 5 exhibited positive connectivity. Particularly, strong positive connectivity is observed between SM and CC and VIS in shared dynamic module 1, as are connections between CC and SUB, and VIS and DMN. These positive interactions among sensorimotor, cognitive control, and visual networks, which are consistent with their roles in integrated sensory processing and attention, are known to be frequently disrupted in schizophrenia in schizophrenia (Kaufmann et al., 2015; Berman et al., 2016). In dynamic module 2, the connection between DMN and SM shows an extremely strong positive correlation, alongside strong positive connections between CC and SUB, CC and SM, and DMN with CC and VIS. The involvement of the DMN with task-positive networks in this module may reflect a functional balance between internally and externally oriented cognition, the alteration of which has been linked to symptom severity in schizophrenia (Calhoun and Adali, 2012). In shared dynamic modules 3 and 4, negative connections between components are dominant, especially in dynamic module 3 where all components show negative connectivity. Module 4 displays weak positive values between CB and other ICNs, while negative connections are evident between VIS and AUD, SM, CC, and DMN. The predominance of negative connectivity in these modules, particularly involving the default mode and visual networks, may represent a mechanism for suppressing task-irrelevant activity, and its disruption could contribute to cognitive disorganization and sensory processing deficits observed in patients (Kuang et al., 2024). In dynamic module 5, DMN shows strong positive connectivity with other ICNs, especially between SUB and CC. This pattern underscores the integrative role of the DMN with subcortical and cognitive control regions, supporting high-level functions such as memory and self-referential thought, which are frequently impaired in schizophrenia (Yamamoto et al., 2022). Aberrant connections across SM, CC, VIS, AUD, SUB, and DMN have been consistently implicated in schizophrenia pathology, as supported by previous investigations (Kuang et al., 2024; Calhoun and Adali, 2012; Kaufmann et al., 2015; Berman et al., 2016; Yamamoto et al., 2022).

Second, as illustrated in Figure 3, SM and CC exhibit higher *Ai*_*r*_(*icn*_1_) values and *p*_*r*_(*icn*_1_) values than other ICNs in shared dynamic modules 1 and 2, indicating that SM and CC play a core role in the brain functional network in these two modules. AUD and CB exhibit very low *Ai*_*r*_(*icn*_1_) values in all 5 dynamic modules, which are caused by both positive and negative connectivity values within AUD and CB as shown in Supplementary material. In particular, AUD has negative *p*_*r*_(*icn*_1_) values in shared dynamic modules 2, 3, and 4, while CB has negative *p*_*r*_(*icn*_1_) values in shared dynamic modules 2 and 3. This pronounced variability in within-ICN connectivity suggests that auditory and cerebellar networks are particularly susceptible to disruption in schizophrenia. Auditory network instability may underlie the generation of auditory hallucinations, a core symptom of the disorder (Rashid et al., 2014). Similarly, cerebellar involvement in cognitive and motor coordination is well-documented, and its fluctuating connectivity patterns could contribute to the cognitive dysmetria and motor abnormalities frequently observed in patients (Han et al., 2023). It has been also demonstrated that schizophrenia shows abnormalities in the functional connectivity of certain brain areas, which may affect the connectivity strength between AUD and CB (Han et al., 2023; Rashid et al., 2014).

As shown in Figure 2, most of the between-ICN connections of HCs are negative in shared dynamic module 2, and the between-ICN connections in various frequency ranges are also analyzed. In fact, the majority of the energy of time courses extracted by group ICA concentrate on low-frequency band 1 (as shown in Supplementary material) which is primarily distributed in states 2 and 3. The supplementary energy of time courses spread on high-frequency band 3 which is primarily in state 3. While the lower energy of time courses scatter on the medium-frequency band 2 which is primarily concentrated in states 1 and 2. This implies that different frequencies affect the transition probability among the three states. In addition, frequency bands 1 and 3 which conclude the majority energy of time courses, present lower FT values than frequency band 2 as shown in Figure 6. In frequency bands 1 and 2, the FT values of the HC and SZ groups are very close, however, in frequency band 3, the FT values of SZs and HCs show a large change for the state that exhibits the highest FT in that band. This suggests that the SZ group spends more fractional time in the dominant high-frequency state. At frequency band 2, DT and FT values in state 2 exhibit opposite tendencies between HCs and SZs. This suggests that the FT of the SZs remaining in state 2 at this frequency band is greater, but its average stay time in state 2 is smaller. In reality, the states with the highest FT and DT values—specifically, state 3 in frequency band 1, state 2 in frequency band 2, and state 3 in frequency band 3—also show higher values in state transition matrices. These frequency-specific states may have a certain 'stickiness' or stability (Luo et al., 2020). Moreover, compared to HCs, SZs display higher DT and FT values in the dominant states of frequency bands 1 and 3 (i.e., state 3 in band 1 and state 3 in band 3). This indicates that the stability of these frequency-specific neuronal states in SZs may contribute to cognitive impairments, including hallucinations, delusions, distractibility, memory loss, and confusion in speech or thought (Luo et al., 2020; Nyatega et al., 2021). As found in Miller et al. (2016), SZs obtain lower NT values than HC in all frequency bands, demonstrating the low frequency of state switching and lower flexibility of the SZ group. The above results of dynamic connections and the time-frequency state analysis can provide more comprehensive information on brain functional connections than traditional dFNC analysis and gain a deeper understanding of brain dynamics and cognition.

It is important to recognize that there are certain restrictions. For instance, many states did not achieve statistically significant differences between HCs and SZs. Other tensor decomposition approaches, such as Tucker decomposition, offer complementary strengths. Han et al. (2024) applied Tucker decomposition to extract shared and subject-specific spatial-temporal information, while Gabrielson et al. (2024) recently proposed a mode coresets-based Tucker method that enables efficient feature selection in fMRI analysis. These Tucker decomposition methods could be explored in future work to either capture more complex multi-way interactions or to expand into coupled Tucker decomposition based on our method, potentially enhancing both interpretability and computational efficiency in multi-frequency dFNC studies.

## Data Availability

Publicly available datasets were analyzed in this study. This data can be found here: http://fcon_1000.projects.nitrc.org/indi/retro/cobre.html.
