# Supplementary material for: CCPD under sparsity and low-rank constraints: multi-frequency dynamic functional network connectivity analysis in schizophrenia
Source: Front Neurosci. 2026 May 8;20:1775687. doi: 10.3389/fnins.2026.1775687 (PMC13194389; doi:10.3389/fnins.2026.1775687)
Supplement: Supplementary file 1 [file Supplementary_file_1.pdf]

Supplementary Material

S1. 41 INDEPENDENT COMPONENTS EXTRACTED BY GROUP ICA

A total of 100 ICs were extracted by group ICA and then 41 interesting ICs are selected for further processes. The detailed shared spatial maps of 41 ICs were displayed in Figure S1, which are further divided into seven ICNs: subcortical (SUB, 8 ICs), auditory (AUD, 2 ICs), somatomotor (SM, 9 ICs), visual (VIS, 6 ICs), cognitive control (CC, 9 ICs), default mode network (DMN, 5 ICs), and cerebellar (CB, 2 ICs).

S2. INTACT CONNECTION TOPOLOGIES OF 5 SHARED DYNAMIC MODULES

According to the shared dynamic connectivity loading matrix A extracted by the proposed method, the symmetric shared dynamic connectivity matrix of each module is obtained by transforming each vector of A. Therefore, the intact connectivity topologies of all 41 component of five shared dynamic modules are shown in Figure S2.

S3. SPECTRUM DIAGRAM OF THE TIME COURSES EXTRACTED BY GROUP ICA

The time courses and spatial maps of 145 subjects are obtained by group ICA in this paper. By performing fast Fourier transform on all time courses of all subjects, the spectrum diagram of the time courses is shown in Figure S3. As dislayed in Figure S3, there is the largest amplitude in the low-frequency band samller than 40 Hz, the amplitude gradually decreases with the increase in frequency, and a larger amplitude in higher band ranging from 100 Hz to 130 Hz. As such, we divide it into 3 frequency bands: low-frequency band 1: 0-25 Hz, medium-frequency band 2: 25-50 Hz, and high-frequency band 3: 100-130 Hz. It can be seen that most of the energy of the time courses concentrates in low-frequency band 1, supplementary energy is distributed in high-frequency band 3, and lower energy is scattered in medium-frequency band 2.

S4. MODEL ORDER SELECTION FOR THE PROPOSED SLRCCPD METHOD

To determine the optimal number of dynamic connectivity modules R, we systematically evaluated R values ranging from 3 to 7 using the proposed SLRCCPD framework. For each R, we computed three performance metrics: the goodness-of-fit (GoF, mean $\pm$ standard deviation across five runs), the number of significantly different dynamic module pairs (SDP) ($p < 0.05$), and clustering stability (measured by the Adjusted Rand Index across ten k-means runs on the subject-specific time-frequency weights). The results are summarized in Table S1.

S5. PAIRED T-TEST RESULTS FOR CONNECTION-LEVEL GROUP DIFFERENCES IN DYNAMIC MODULES

To directly assess the statistical significance of the visual patterns in Figure 4, paired t-tests were performed on the 820-dimensional between-ICN connectivity vectors of each dynamic module, using the group-averaged connectivity matrices $\mathbf{A}_{\mathrm{HC}}$ and $\mathbf{A}_{\mathrm{SZ}}$ derived from equation (22). The resulting p-values were corrected for multiple comparisons using the false discovery rate (FDR). The subject-specific

connectivity loading weights of HCs and SZs $\mathbf{A}_r^{(1)} = \{\mathbf{a}_k^{(1)}\} \in \mathbb{R}^{I \times K_1}$, and $\mathbf{A}_r^{(2)} = \{\mathbf{a}_k^{(2)}\} \in \mathbb{R}^{I \times K_2}$, of each dynamic module are calculated as follow:

$$\mathbf{A}_r^{(1)} = \mathbf{a}_r \mathbf{c}_r^{(1)T}, \quad \text{and} \quad \mathbf{A}_r^{(2)} = \mathbf{a}_r \mathbf{c}_r^{(2)T}.$$

The paired t-tests were also performed on arbitrary $\mathbf{a}_k^{(1)}$ $(k = 1, \ldots, K_1)$ and $\mathbf{a}_k^{(2)}$ $(k = 1, \ldots, K_2)$, and the number of significant connections ($p < 0.05$) was counted for each dynamic module. Table S2 reports the p-value, FDR-adjusted q-value, and t-value for each module. Modules 2, 3 and 4 show significant differences between HC and SZ (FDR-corrected q <0.05) and large number of significant connections, confirming that the pronounced color differences observed in Figure 4 are statistically supported.

S6. THE STATICAL GROUP DIFFERENCE RESULTS OF STATE CHARACTERISTIC INDICATORS

The frequency-band specific state characteristic indicators including FT, DT, and NT values are derived from the subject-specific time-frequency weight matrices $\mathbf{H}_{\mathrm{HC}}^{(f)}$ and $\mathbf{H}_{\mathrm{SZ}}^{(f)}$, as shown in Figure 6. The statistical comparisons between HC and SZ groups were performed using Mann-Whitney U tests. Table S3 reports the p value and effect size r (rank-biserial correlation) for each indicator at each band and each state.

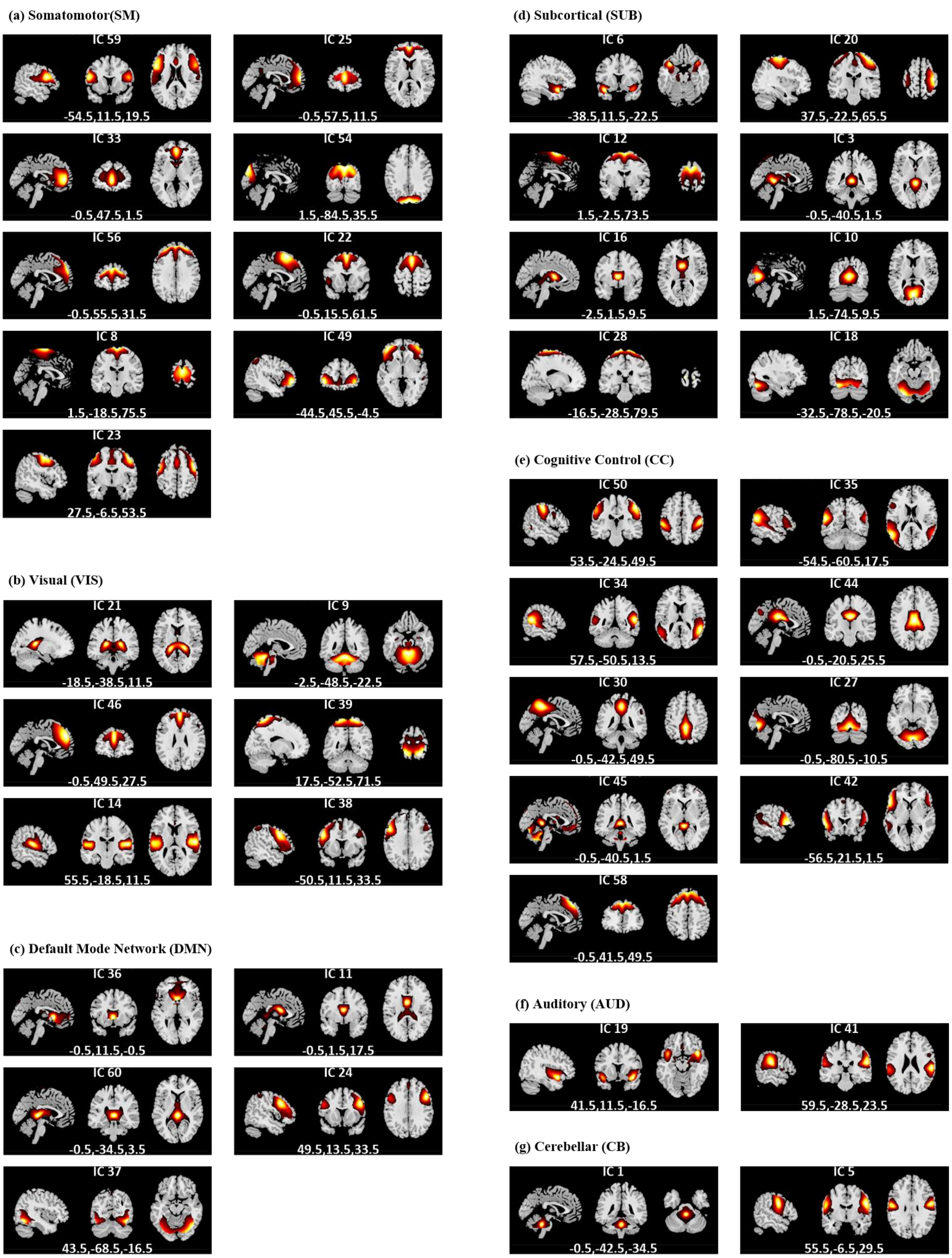

Figure S1. The shared spatial maps of 41 ICs extracted by GICA which are further sorted as seven ICNs.

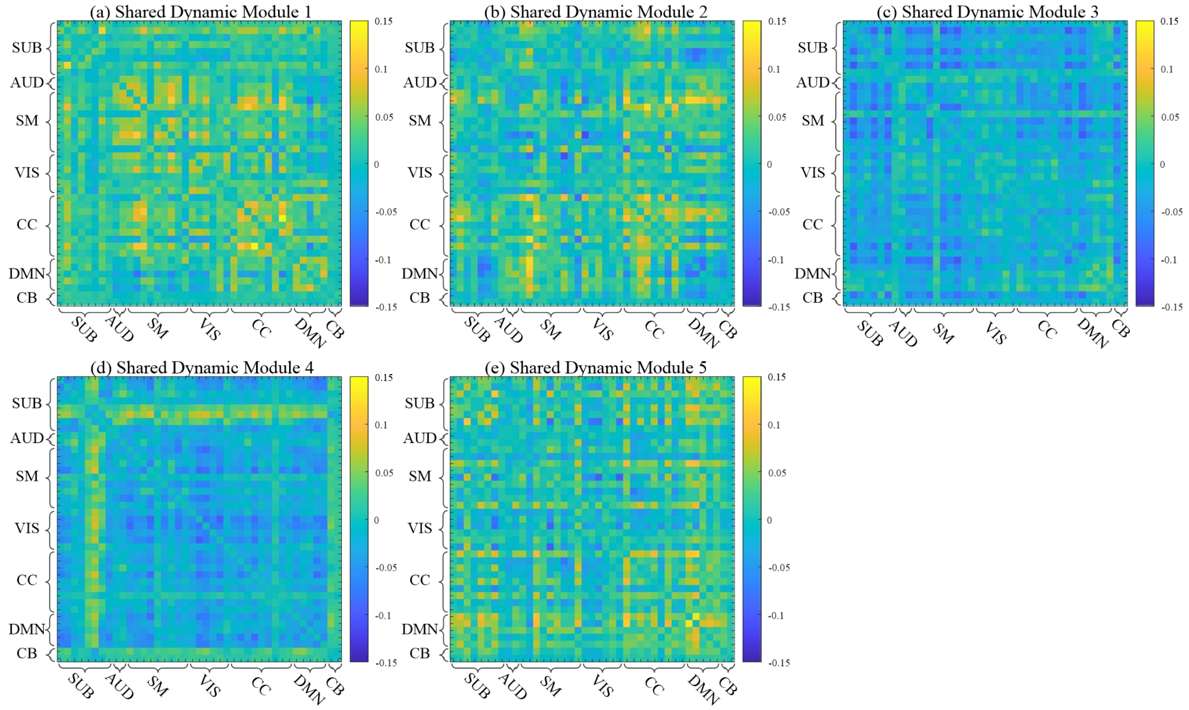

Figure S2. Intact connection topologies between all 41 ICs in five shared dynamic modules.

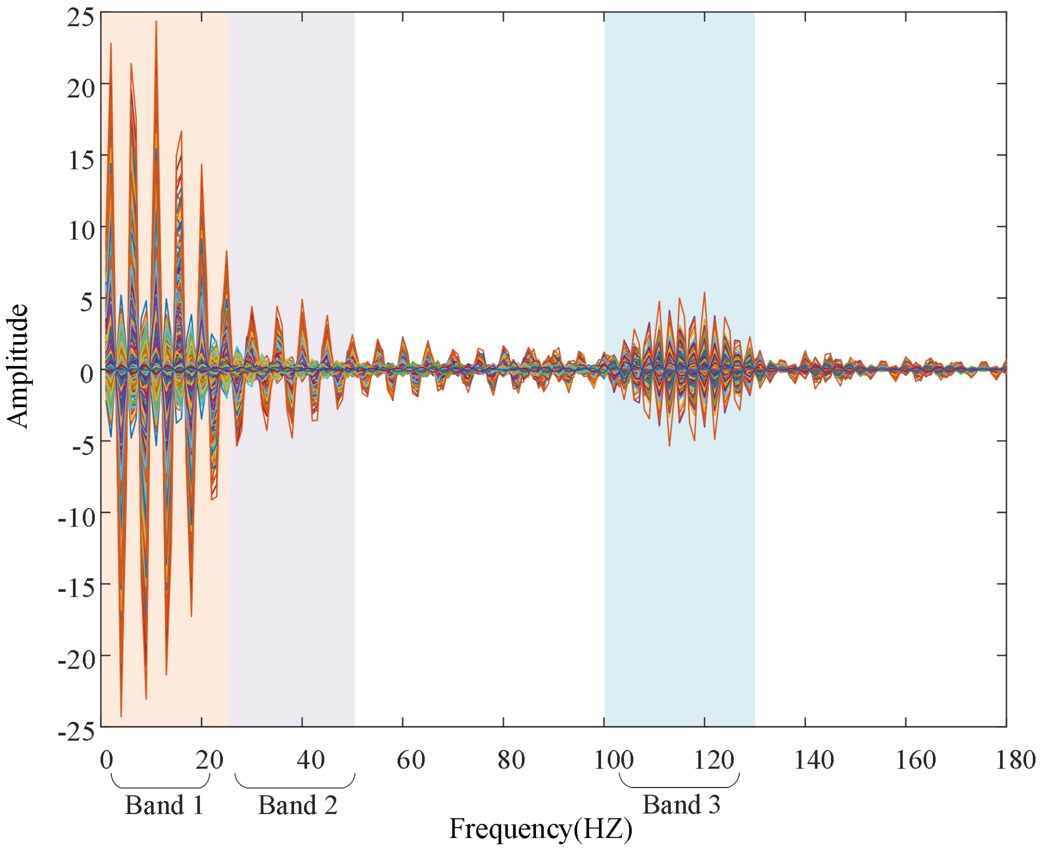

Figure S3. Spectrum digram of the time courses extracted by group ICA.

Table S1. Model order R effect of the proposed SLRCCPD evaluated by goodness-of-fit (GoF, mean $\pm$ std), the number of significantly different module pairs (SDP), and clustering stability.

R	**3**	**4**	**5**	**6**	**7**
GoF	0.089 ± 0.000	0.102 ± 0.000	0.114 ± 0.001	0.125 ± 0.001	0.135 ± 0.002
SDP	2	5	16	9	12
Clustering stability	0.865	0.921	0.765	0.686	0.673

Table S2. Paired t-test results for the between-ICN connectivity difference and the number of significant different connections between HCs and SZs in each dynamic module.

Module	p value	q value (FDR)	t value	Significant connections
1	7.8653e-01	7.8653e-01	-0.27	12
2	1.9938e-04	9.9690e-04	-4.09	124
3	8.0334e-04	2.0084e-03	3.62	98
4	1.0472e-02	1.7453e-02	-2.68	156
5	4.3564e-01	5.4455e-01	0.79	8

Table S3. The Mann-Whitney U tests of state characteristic indicators (FT, DT, and NT values) at each frequency band. The p value and effect size r value are given.

		Band 1		Band 2		Band 3	
State	Measure	p value	r value	p value	r value	p value	r value
1	FT	0.9632	0.004	0.0001	0.315	0.2928	0.093
	DT	0.7750	-0.024	0.0021	0.217	0.5496	0.053
2	FT	0.0038	-0.157	0.0015	0.243	0.2731	-0.079
	DT	0.0027	-0.213	0.0022	-0.234	0.2496	-0.083
3	FT	0.0099	0.273	0.3299	-0.097	0.0002	0.285
	DT	0.0066	0.276	0.3076	-0.077	0.0002	0.287
	NT	0.0005	-0.058	0.0003	-0.301	0.0006	-0.281
